# Consumption, Attitudes, and Trends of Vending Machine Foods at a University Campus: A Cross-Sectional Study

**DOI:** 10.3390/foods10092122

**Published:** 2021-09-08

**Authors:** Hayder Hasan, Moez Al-Islam E. Faris, Maysm N. Mohamad, Ayesha S. Al Dhaheri, Mona Hashim, Lily Stojanovska, Rameez Al Daour, Malak Rashid, Lena El-Farra, Azza Alsuwaidi, Heba Altawfiq, Zainab Erwa, Leila Cheikh Ismail

**Affiliations:** 1 Department of Clinical Nutrition and Dietetics, College of Health Sciences, University of Sharjah, Sharjah 27272, United Arab Emirates; haidarah@sharjah.ac.ae (H.H.); mfaris@sharjah.ac.ae (M.A.-I.E.F.); mhashim@sharjah.ac.ae (M.H.); rameezdaour@hotmail.com (R.A.D.); U16100647@sharjah.ac.ae (M.R.); U16103270@sharjah.ac.ae (L.E.-F.); U15100360@sharjah.ac.ae (A.A.); U15106336@sharjah.ac.ae (H.A.); U16103580@sharjah.ac.ae (Z.E.); 2 Department of Nutrition and Health, College of Medicine and Health Sciences, United Arab Emirates University, Al Ain 15551, United Arab Emirates; drmaysm@gmail.com (M.N.M.); ayesha_aldhaheri@uaeu.ac.ae (A.S.A.D.); lily.stojanovska@uaeu.ac.ae (L.S.); 3 Institute for Health and Sport, Victoria University, Melbourne 14428, Australia; 4 Nuffield Department of Women’s & Reproductive Health, University of Oxford, Oxford OX1 2JD, UK

**Keywords:** beverages, consumer, food environment, snacks, vending machine

## Abstract

Vending machines (VMs) have been identified as an obesogenic factor, offering mainly energy-dense and nutrient-poor foods, with limited healthy options available. This cross-sectional study aimed to assess consumption trends and attitude toward vending machine (VM) foods in a university setting. A web-based survey was conducted among 1250 students and staff. Most participants reported weight gain (43.4%) and poorer food choices (53.4%) since joining the university. Participants described VM foods as expensive (53.7%) and lacking variety (34.3%). Over 81% demanded the availability of healthier options. About 75% of participants were VMs users. The most frequently purchased VM items were water, chocolate, and chips. Males reported consuming nuts, soda, iced tea, and energy drinks more frequently than females (*p* < 0.005). The main reasons for using the VM were hunger and lack of time. Over 40% nominated fresh fruits, baked chips, sandwiches, and dry roasted nuts to be provided in the VMs as healthier food options. Males and those responsible for buying their own food were more likely to select healthier options (*p* = 0.001). Findings can be used to inform stakeholders of current vending behaviors and to plan tailored interventions to improve the nutritional quality of vended items and promote healthier food choices.

## 1. Introduction

Globally, obesity has tripled since 1975 [1], and data in 2016 showed that 39% of adults aged 18 years and over were overweight, and 13% were obese [2]. In the United Arab Emirates (UAE), overweight and obesity have risen dramatically over the past decade [3]. Obesity is now considered a major health problem in the UAE community and it is a major risk factor for non-communicable diseases (NCDs) such as cardiovascular disease, diabetes mellitus, musculoskeletal disorders, and some cancers [4]. A recent study among university students in the UAE revealed that over one-third of students were classified as overweight or obese and 6.8% had metabolic syndrome [5]. Many factors contribute to excessive body weight such as genetics, environmental factors, social background, and physical activity level [6].

The food environment of universities was recognized by the World Health Organization as an important health-promotion setting [7]. It has an influence on faculty, staff, and students, who are young adults during a critical transitional and developmental phase. Therefore, universities could potentially contribute to creating healthy working and learning environment through their policies and practices. However, the food environment in universities offers largely unhealthy food and beverages over healthier options, including through vending machines (VMs). A recent study in the UAE assessed the nutritional value of snacks and beverages in VMs at four university campuses and revealed that 65% of them were calorie-dense and offered a high content of sugar, sodium, and saturated fat [8]. Moreover, a series of recent studies have indicated that foods and beverages sold in the VMs tend to be low in nutritional value and high in calories, fat, salt, and sugar [9,10,11].

VMs provide a convenient and ready source for a range of foods and beverages. However, VM accessibility has been positively associated with higher consumption and greater frequency of snacks [12]. As a result, the frequent use of VM foods has been associated with the development of an obesogenic food environment at universities [13]. In a qualitative interview study in the UAE, participants indicated the need to improve the nutritional quality of the food items sold in the campus VMs in addition to placing nutrition guidelines on these service tools [14].

A systematic review of VM nutrition interventions at university settings concluded that effective interventions involve providing healthier VM replacement, reducing the prices of healthier items, and promoting them through awareness campaigns [15]. Increasing the availability of healthy products with and without additional nutrition communication at a university in Italy nudged the consumers’ food choices towards healthy options [16]. Hua et al. concluded that the promotion of healthier VMs food options had a small effect on sales volume; however, pairing it with the availability of healthier items had a significantly higher impact [17]. Additionally, a review by Grech et al., found that the most effective strategies to increase the sales of healthier items were increasing the availability of healthier options and reducing their prices [18].

However, informing stakeholders of the need to improve the availability and price of healthy foods vended at the university would not be enough. As there are currently limited data in the UAE on the frequency of consuming vended foods at universities, and the attitudes and behaviors regarding vending options. Therefore, to lay the foundation for intervention studies, this study aimed to assess the current food choices and the likeliness of selecting healthier items if they were provided at the university VMs.

## 2. Materials and Methods

### 2.1. Study Design and Participants

A cross-sectional design was applied, and an electronic self-administrated questionnaire was adapted from the literature and used to assess the attitudes and trends of consumption from VM foods and beverages available at the UOS campuses [19,20]. The target population included UOS students, staff, and faculty members. These were invited to participate in an online survey using a snowball sampling method to guarantee a large-scale distribution and recruitment of participants. A total of 1250 participants (23.7% of males) were included in this study.

A URL web link was retrieved for the survey and was distributed using invitations through the internal e-mail system and social media platforms of the university. All participants provided an electronic consent form before participation and were given the right to withdraw at any point during the survey. Consenting participants then proceeded to complete and submit their responses. All data were collected anonymously with no indication of any personal information and participants were not rewarded. The questionnaire required 10 min to complete and included a screening question to exclude participants who were not students, staff, or faculty members at UOS.

The present study followed the ethical code for web-based research [21,22] and conforms to the principles embodied in the Declaration of Helsinki [23]. The study protocol was approved by the Research Ethics Committee at the University of Sharjah (REC-20-05-13-03-S).

### 2.2. Survey Questionnaire

A multicomponent, self-administrated online survey was designed using Google Forms in English. This questionnaire was divided into three main sections: demographics, frequency of consumption, and attitudes.

#### 2.2.1. Demographic Information

This section included questions about participants’ sex, age, nationality, university position, department of study/work, and living arrangement. This section also included questions on the responsibility of food shopping and food preparation; whether there was a change in body weight since joining the university for work or study; and if eating habits were affected since their enrolment. The last question in this section inquired about the frequency of using VMs with three answer options: “I do not use vending machines”, “1–2 times per week”, or “>3 times per week”. Participants who answered the last question as “I do not use vending machines” were automatically directed to the third section of the survey on the attitudes toward VMs and skipped the second section about the frequency of consuming VM products.

#### 2.2.2. Frequency of Consumption

Participants who reported using VMs were asked to specify the frequency of purchasing different foods and drinks available at the VMs with three response options “0 times per week”; “1–2 times per week”; or “>3 times per week”. The products available in the VMs around campus were assessed and classified into food categories including five foods: chocolate bars/wafers, chips, cookies/biscuits, candies, and nuts; and beverage category including six foods: soda, iced tea, energy drinks, flavored milk, fruit juice, and water. The users of the VMs were also asked about the reasons for purchasing products from the VM, and the responses included hunger, convenience, lack of time, snacking between meals, or other reasons.

#### 2.2.3. Vending Machine Attitudes

The last section examined the opinions of participants regarding VM products. Participants were asked to report if they thought VMs offered a diverse enough range of products; if they would like to have healthier food and drink choices in the VMs, and if they think VM products are expensive. Answer options for the four questions were “agree”; “neutral”; or “disagree”. Moreover, participants were asked to rate how likely they were to purchase a list of healthy food and beverages if provided in the VMs (e.g., fresh salads, fresh vegetables, fresh fruits, popcorn, raw nuts, baked chips) with three response options “likely”; “neutral”; or “unlikely”.

### 2.3. Data Analysis

Categorical variables were summarized as counts and percentages. A Chi-square test was used to determine the association between sex and frequency and attitudes of consumption from the VM. Each item of the suggested healthy options was scored 1 if the response to it was “likely” to purchase or 0 if the response was “neutral” or “unlikely” to purchase the food option. All items were summed, and the total likeliness score was obtained (minimum = 0 and maximum = 15), with a higher score indicating a higher likeliness of buying healthier options. To find the best predictor for the likeliness of purchasing healthier options the linear regression analysis was used. Results were considered significant for *p*-value < 0.05. Statistical analysis was performed using Statistical Package for the Social Sciences (SPSS) version 26.0 (IBM, Chicago, IL, USA).

## 3. Results

### 3.1. Sociodemographic Characteristics

The sociodemographic characteristics of the study population are presented in Table 1. The male-to-female ratio was almost 1:3, with 23.7% males. The majority (73%) of surveyed individuals were aged 18–24 years, more than half (51.8%) were Arab residents, about three-fourths (74.5%) were undergraduate students, 39.9% were working or studying in the medical field, about three-fourths (75.2%) were living outside the dorms, 60.6% were relying on family members for food shopping and more than half (56.7%) were relying on family members for food preparation. The majority of participants reported weight gain and poorer food choices since joining the university for work or study (43.4% and 53.4%, respectively). Three-fourths (75%) of participants reported using the VMs at least once per week and the rest (25%) of the participants did not report using the VMs.

### 3.2. Frequency of Consumption

Only those who reported using the VMs (*n* = 937) were asked questions on the frequency of consuming food and beverages from the machines (Table 2). The majority of VM users purchased chocolate bars/wafers (56.9%) and chips (40.0%) at least once per week. However, over half of the study participants did not purchase the following items from the VMs: cookies/biscuits (60%), candies (66.5%), nuts (66.7%), soda (77.5%), iced tea (83.9%), energy drinks (87.5%), flavored milk (77.1%) and fruit juice (60.1%). Over 91% of the participants purchased water from the VMs at least once per week.

There was a difference between males’ and females’ frequency of consumption for nuts (*p* = 0.019), soda (*p* < 0.001), iced tea (*p* = 0.004), and energy drinks (*p* = 0.004). The frequency scores suggest that males purchase these foods and beverages more frequently than females.

**Figure 1:**
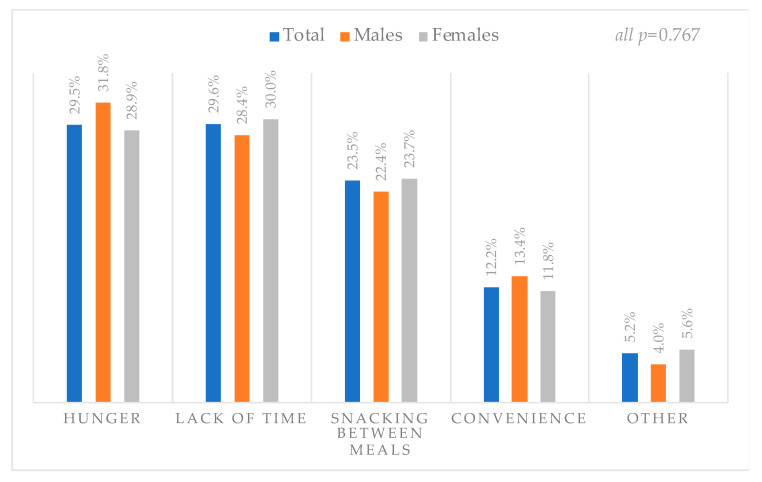
Reasons for purchase from the vending machine among users (*n* = 937). The *p*-value indicates the statistical significance of the Chi-square test.

Among VM users, the most common reasons for purchase from the VMs were hunger/thirst (29.5%), lack of time (29.6%), and snacking between meals (23.5%) (Figure 1). Most males purchased from the VMs due to hunger/thirst (31.8%) while the majority of females used the VMs because of lack of time to visit the canteen (30.0%) with no statistical difference between sexes.

### 3.3. Vending Machine Attitudes

Only 21.6% of the participants agreed that there was a variety of food items available in the VMs (Table 3). The majority of participants preferred having a healthy snack and drink options provided in the VMs (81.8%, and 85%, respectively). More than half of the participants (53.6%) agreed that food items provided in the VMs were expensive, more males compared to females (*p* = 0.045).

Table 4 displays the participants’ likeliness to buy healthy food items if introduced in the VMs. The majority of participants nominated fresh fruits (45.6%), baked chips (43.8%), sandwiches (42.6%), dry roasted nuts (41.5%), and dark chocolate (36.7%) as very likely to buy food items. In contrast, most participants reported that they were unlikely to buy plain milk (47.1%), fresh vegetables (44.6%), dried fruits (41.3%), frozen yogurt (39.5%), protein bars (37.7), and plain yogurt (36.2%) if provided in the VMs.

Male participants reported that they were very likely to purchase 100% fresh fruit juice (39.5%), dry roasted nuts (38.2%), and fresh fruits (37.8%) if offered. On the other hand, they were unlikely to buy fresh vegetables (49.3%), frozen yogurt (49.3%), dried fruits (37.5%), and protein bars (37.5%). Female participants were willing to purchase baked chips (48.1%), fresh fruits (48.0%), and sandwiches (44.4%) and were unlikely to buy, fresh vegetables (43.2%) and dried fruits (42.5%). Moreover, the results in Table 3 also demonstrate that both sexes were unlikely to buy plain milk and protein bars. However, there was a difference between sexes in the likeliness of purchasing fresh fruits (*p* < 0.001), plain yogurt (*p* = 0.007), popcorn (*p* < 0.001), sandwiches (*p* = 0.049), frozen yogurt (*p* < 0.001), and baked chips (*p* < 0.001).

Table 5 demonstrates the association of different variables with the likeliness of purchasing healthier options. Males seem to be more likely to purchase healthier items (B = −0.95, *p* = 0.001, 95% CI: −1.53–−0.37). Similarly, if the participant is purchasing for him/herself, gaining weight, more frequently using the VM and purchasing from the VM for non-hunger reason are more likely to purchase healthier items (B = 1.25, *p* < 0.001, 95% CI: 0.74–1.76, B = 0.39, *p* = 0.006, 95% CI: 0.11–0.66, B = 0.49, *p* = 0.049, 95% CI: 0.01–0.97, and B = −0.69, *p* = 0.009, 95% CI: 0.17–1.21 respectively). Moreover, the best predictor for the likeliness of purchasing healthier options was when the person is purchasing for oneself (B = 1.25, *p* < 0.001, 95% CI: 0.74–1.76).

## 4. Discussion

The current study aimed to assess the attitudes and trends of consumption of VM food items at the UOS via an electronically self-administrated questionnaire. The results suggested that more than half of the participants relied on family members for food preparation, and lack of cooking skills has previously been suggested as a barrier to conducting healthy shopping and making healthful food choices [24]. The majority of participants indicated that they gained weight and had worse eating habits since joining the university. A recent systematic review of weight change among college students suggested that approximately 30% of them gained more than 4 kg [25]. Moreover, a study among United States college students indicated that the diet of the students was influenced by food choices in their surrounding environment [26]. Health problems and weight gain among young adults may be due to low consumption of fruits and vegetables, high consumption of junk food, irregular meals, poor snacking behavior, and breakfast skipping [27]. Moreover, a recent review identified main obesogenic environmental factors including physical inactivity, screen-related immobility, imbalanced diet, portion sizes, speed eating, and the availability of junk food and sweet drinks at low prices [28]. In a study assessing the quality of snacks and beverages sold in the UOS VMs, the majority of vended food items were calorie-dense and none of them met the criteria of low in sugars or high in fiber [8,29]. Researchers indicated that lack of time, high food prices, less availability of healthy options, and lack of motivation were the most common barriers to healthy eating among students [29,30].

The results showed that the main reasons for purchasing from VMs were hunger and lack of time to visit the canteen. These findings are similar to the results of other studies where the motivations for purchase were mostly influenced by hunger, convenience, time, and prices of VM foods [31,32]. Notably, VMs were mostly used among the study participants to purchase water, chocolate bars/wafers, and chips. This is in support of earlier research where VM users purchased chocolate bars and crisps more than other snacks and bought water and regular soft drinks more than other drinks [20]. Similarly, a study in the United States revealed that the most consumed VM items were chips, crackers, candy bars, soda, and sports drinks [33]. The results of our study indicate that males consume more soda and energy drinks compared to females. Similarly, a recent cross-sectional study among college students in Jordan revealed that male students consumed significantly more calories from sugar-sweetened beverages compared to female students [34].

One main finding of this study suggests that consumers were willing to purchase healthier choices if they were available. Current understanding supports the importance of providing healthier options to improve dietary choices. A study on university students and hospital employees showed that most participants from both a university campus and a public hospital considered foods currently available in VMs as “too unhealthy” and they were interested in a range of healthier snacks to be available in VMs [35]. A similar conclusion was drawn from a study conducted in the UAE where students had a strong preference towards nutritious options, contrary to what was offered in the VMs [14].

Participants were interested in purchasing fresh fruits, baked chips, sandwiches, popcorn, and dry roasted nuts from the VMs. This is consistent with what has been found in previous research that suggested an incline in the number of students buying fresh fruits [14]. On the other hand, participants were not likely to purchase plain milk, fresh vegetables, and dried fruits. This was previously reported that college students were less likely to purchase milk from VMs [36]. The findings of the current study revealed that males were more likely to purchase healthier options if they were provided in the VMs. On the contrary, consumer research reported that female customers expressed a stronger wish for healthier options and were more likely to make healthier choices [37,38]. Our data suggest that users of VMs were more likely to buy healthier food options; this indicates that they are aware of the low nutritional quality of vended foods and might make better choices if environmental changes were implemented to increase offerings of more nutrient-dense options. Moreover, the findings of this study revealed that the best predictor of purchasing healthier options was having the responsibility of doing household grocery shopping. This is in support of earlier research among Slovenian consumers, which showed that those responsible for household grocery shopping were significantly more familiar with healthier food options [39].

Such findings are important when planning interventions at the university and community level and could be used to make informed decisions with the supplying companies. An intervention study in a community health organization concluded that increasing the percentage of healthier options in VMs significantly reduced the amounts of calories, sodium, fats, and sugars vended, without having a negative financial impact or reducing the number of vended items [40]. Moreover, a systematic review showed that intervention studies were effective at increasing sales of healthier food choices by reducing their prices or increasing their availability [41].

There are limitations to the present study. The use of a self-reported questionnaire might have led to misreporting of data. Additionally, conducting the study in one university and using a convenience sampling technique limits the generalizability of the results to other settings in the UAE. However, the findings of the current study can be indicative of consumer attitudes and trends of VM foods and thus could provide intervention opportunities for institutes providing products in the VMs. Attention should be given to making changes in food options available in the VMs to improve the consumer’s choices and overall health. Offering healthy food options in the VMs should be taken into consideration especially that most of the participants were willing to purchase healthier food items if provided. Moreover, providing nutrition education may also guide consumers in making healthier choices.

## Figures and Tables

**Table 1:** Sociodemographic characteristics of the participants (*n* = 1250).

Variable	*n*	%
Sex		
Male	296	23.7
Female	954	76.3
Age group (years)		
18–24	913	73
25–34	161	12.9
35–44	96	7.7
>45	80	6.4
Nationality		
Local citizen	383	30.6
Resident, Arab	647	51.8
Resident, Non-Arab	220	17.6
University position		
Undergraduate student	931	74.5
Graduate student	94	7.5
Staff	162	13
Faculty	63	5
Field of study/work		
Medical ^1^	499	39.9
Applied Sciences ^2^	402	32.2
Humanities ^3^	233	18.6
Other	116	9.2
Living arrangement		
Dorms	310	24.8
Outside dorms	940	75.2
Food shopping responsibility		
Myself	468	37.5
Family member	758	60.6
Friend	4	0.3
Helper	20	1.6
Food preparation responsibility		
Myself	335	26.8
Family member	709	56.7
Friend	3	0.2
Helper	162	13
Buy ready meals only	41	3.3
Bodyweight change since enrollment		
Gained	542	43.4
Lost	293	23.4
Stable	338	27
I do not know	77	6.2
Eating habits change since enrollment		
Better	234	18.8
Worse	668	53.4
No changes	255	20.4
I do not know	93	7.4
Frequency of using vending machines		
I do not use vending machines	313	25
1–2 times per week	588	47
>3 times per week	349	28

^1^ Medical major include health sciences, medicine, dental medicine, and pharmacy. ^2^ Applied sciences include engineering, sciences, and business. ^3^ Humanities include law, Sharia (Islamic studies), social sciences, communication, and fine arts.

**Table 2:** Frequency of consumption from the vending machine among users (*n* = 937).

Frequency of Purchase	Total % (*n*)	Males % (*n*)	Females % (*n*)	*p*-Value *
Chocolate bars/wafers				
0 times/week	37.0 (346)	38.3 (77)	36.5 (269)	0.749
1–2 times/week	51.5 (483)	49.3 (99)	52.2 (384)	
>3 times/week	11.5 (108)	12.4 (25)	11.3 (83)	
Chips				
0 times/week	43.1 (404)	48.8 (98)	41.6 (306)	0.171
1–2 times/week	47.7 (447)	43.8 (88)	48.8 (359)	
>3 times/week	9.2 (86)	7.5 (15)	9.6 (71)	
Cookies/biscuits				
0 times/week	60 (562)	54.2 (109)	61.5 (453)	0.137
1–2 times/week	34.9 (327)	40.8 (82)	33.3 (245)	
>3 times/week	5.1 (48)	5.0 (10)	5.2 (38)	
Candies				
0 times/week	66.5 (623)	65.2 (131)	66.8 (492)	0.191
1–2 times/week	28.9 (271)	27.8 (56)	29.2 (215)	
>3 times/week	4.6 (43)	7.0 (14)	4.0 (29)	
Nuts				
0 times/week	66.7 (625)	58.7 (118)	68.9 (507)	0.019
1–2 times/week	29.6 (277)	35.8 (72)	27.9 (205)	
>3 times/week	3.7 (35)	5.5 (11)	3.2 (24)	
Soda				
0 times/week	77.5 (726)	62.2 (125)	81.7 (601)	0.001
1–2 times/week	16.4 (154)	26.4 (53)	13.7 (101)	
>3 times/week	6.1 (57)	11.4 (23)	4.6 (34)	
Iced tea				
0 times/week	83.9 (785)	76.5 (153)	85.9 (632)	0.004
1–2 times/week	13.0 (122)	18.0 (36)	11.7 (86)	
>3 times/week	3.1 (30)	5.5 (11)	2.4 (18)	
Energy drinks				
0 times/week	87.5 (820)	80.6 (162)	89.4 (658)	0.004
1–2 times/week	9.8 (92)	15.4 (31)	8.3 (61)	
>3 times/week	2.7 (25)	4.0 (8)	2.3 (17)	
Flavored Milk				
0 times/week	77.1 (722)	78.1 (157)	76.8 (565)	0.917
1–2 times/week	19.4 (182)	18.4 (37)	19.7 (145)	
>3 times/week	3.5 (33)	3.5 (7)	3.5 (26)	
Fruit juice				
0 times/week	60.1 (563)	55.2 (111)	61.4 (452)	0.067
1–2 times/week	34.0 (319)	35.8 (72)	33.6 (247)	
>3 times/week	5.9 (55)	9.0 (18)	5.0 (37)	
Water				
0 times/week	8.4 (79)	11.5 (23)	7.6 (56)	0.131
1–2 times/week	31.0 (290)	32.8 (66)	30.4 (224)	
>3 times/week	60.6 (568)	55.7 (112)	62.0 (456)	

* The *p*-values indicate the statistical significance of the Chi-square test.

**Table 3:** Opinions of the study participants about vending machine food items (*n* = 1250).

Variable	Total % (*n*)	Males % (*n*)	Females % (*n*)	*p*-Value *
There is a variety of food items in the vending machines				
Agree	21.6 (270)	20.9 (62)	21.8 (208)	0.949
Neutral	44.1 (551)	44.3 (131)	44.0 (420)	
Disagree	34.3 (429)	34.8 (103)	34.2 (326)	
Vending machines should have healthy snack options				
Agree	81.8 (1023)	78.0 (231)	83.0 (792)	0.152
Neutral	14 (175)	16.9 (50)	13.1 (125)	
Disagree	4.2 (52)	5.1 (15)	3.9 (37)	
Vending machines should have healthy drink options				
Agree	85 (1063)	81.1 (240)	86.3 (823)	0.062
Neutral	11.6 (145)	15.5 (46)	10.4 (99)	
Disagree	3.4 (42)	3.4 (10)	3.4 (32)	
Food items available at vending machines are expensive				
Agree	53.6 (670)	59.8 (177)	51.7 (493)	0.045
Neutral	30.3 (379)	27.0 (80)	31.3 (299)	
Disagree	16.1 (201)	13.2 (89)	17.0 (162)	

* The *p*-values indicate the statistical significance of the Chi-square test.

**Table 4:** Likeliness to purchase healthy options from the vending machine among the study participants (*n* = 1250).

Likeliness to Purchase Healthy Options	Total % (*n*)	Males % (*n*)	Females % (*n*)	*p*-Value *
Fresh salad				
Likely	28.5 (356)	23.6 (70)	30.0 (286)	0.108
Neutral	36.4 (455)	38.9 (115)	35.6 (340)	
Unlikely	35.1 (439)	37.5 (111)	34.4 (328)	
Fresh vegetables				
Likely	23.4 (292)	19.9 (59)	24.4 (233)	0.132
Neutral	32.0 (400)	30.7 (97)	32.4 (309)	
Unlikely	44.6 (558)	49.3 (146)	43.2 (412)	
Fresh fruits				
Likely	45.6 (570)	37.8 (112)	48.0 (458)	0.001
Neutral	36.6 (458)	37.5 (111)	36.4 (347)	
Unlikely	17.8 (222)	24.7 (73)	15.6 (149)	
Dried fruits				
Likely	24.6 (307)	25.7 (76)	24.2 (231)	
Neutral	34.2 (427)	36.8 (109)	33.3 (318)	0.310
Unlikely	41.3 (516)	37.5 (111)	42.5 (405)	
Plain yogurt				
Likely	31.8 (398)	27.0 (80)	33.3 (318)	0.007
Neutral	31.9 (399)	39.2 (116)	29.7 (283)	
Unlikely	36.2 (453)	33.8 (100)	37.0 (353)	
Plain milk				
Likely	22.4 (280)	18.9 (56)	23.5 (224)	0.050
Neutral	30.5 (381)	35.8 (106)	28.8 (275)	
Unlikely	47.1 (589)	45.3 (134)	47.7 (455)	
Popcorn				
Likely	36.2 (452)	22.6 (67)	40.4 (385)	
Neutral	36.7 (459)	40.5 (120)	35.5 (339)	<0.001
Unlikely	27.1 (339)	36.8 (109)	24.1 (230)	
Dry roasted nuts				
Likely	41.4 (518)	38.2 (113)	42.5 (405)	0.070
Neutral	37.3 (466)	42.9 (127)	35.5 (339)	
Unlikely	21.3 (266)	18.9 (56)	22 (210)	
Raw nuts				
Likely	18.4 (230)	15.5 (46)	19.3 (184)	0.347
Neutral	48.5 (606)	50.3 (149)	47.9 (457)	
Unlikely	33.1 (414)	34.1 (101)	32.8 (313)	
100% fresh fruit juice				
Likely	41.2 (515)	39.5 (117)	41.7 (398)	0.692
Neutral	49.7 (621)	50.3 (149)	49.5 (472)	
Unlikely	9.1 (114)	10.1 (30)	8.8 (84)	
Dark chocolate				
Likely	36.7 (459)	34.5 (102)	37.4 (357)	0.065
Neutral	34.6 (433)	40.2 (119)	32.9 (314)	
Unlikely	28.6 (358)	25.3 (75)	29.7 (283)	
Protein bars				
Likely	30.6 (383)	30.7 (91)	30.6 (292)	0.997
Neutral	31.7 (396)	31.8 (94)	31.7 (302)	
Unlikely	37.7 (471)	37.5 (111)	37.7 (360)	
Sandwiches				
Likely	42.6 (533)	36.8 (109)	44.4 (424)	0.049
Neutral	33.9 (424)	35.8 (106)	33.3 (318)	
Unlikely	23.4 (293)	27.4 (81)	22.2 (212)	
Frozen yogurt				
Likely	33.4 (418)	21.3 (63)	37.2 (355)	<0.001
Neutral	27 (338)	29.4 (87)	26.3 (251)	
Unlikely	39.5 (494)	49.3 (146)	36.5 (348)	
Baked chips				
Likely	43.8 (548)	30.1 (89)	48.1 (459)	<0.001
Neutral	36.7 (459)	41.9 (124)	35.1 (355)	
Unlikely	19.4 (243)	28 (83)	16.8 (160)	

* The *p*-values indicate the statistical significance of the Chi-square test.

**Table 5:** Linear regression analysis using the likeliness of purchasing healthier options as a dependent variable.

Variable	B	*p*-Value	95.0% Confidence Interval	
			Lower Bound	Upper Bound
Sex	−0.95	0.001	−1.53	−0.37
Food shopping responsibility	1.25	<0.000	0.74	1.76
Food preparation responsibility	0.10	0.409	−0.13	0.32
Bodyweight change since enrollment	0.39	0.006	0.11	0.66
Eating habits change since enrollment	−0.28	0.070	−0.58	0.02
Frequency of using vending machines	0.49	0.049	0.01	0.97
Reasons for purchase from the vending machine	0.69	0.009	0.17	1.21

Coding: Sex: Male = 0; Female = 1. Food shopping responsibility: Myself = 1; Others = 0. Bodyweight change since enrollment: Lost weight = 0; No change = 1; Gained weight = 2. Frequency of using vending machines: I do not use = 0, 1–2 times/week = 1; >3 times/week = 2. Reasons for purchase from the vending machine: Hunger = 0; Others = 1.

## Data Availability

The data that support the findings of this study are openly available in Figshare at https://doi.org/10.6084/m9.figshare.14740797.v1, accessed on 6 June 2021.
